# Age-induced changes in anti-tumor immunity alter the tumor immune infiltrate and impact response to immuno-oncology treatments

**DOI:** 10.3389/fimmu.2023.1258291

**Published:** 2023-10-18

**Authors:** Suzanne I. Sitnikova, Jennifer A. Walker, Laura B. Prickett, Michelle Morrow, Viia E. Valge-Archer, Matthew J. Robinson, Robert W. Wilkinson, Simon J. Dovedi

**Affiliations:** ^1^Early Oncology Discovery, R&D, AstraZeneca, Cambridge, United Kingdom; ^2^Early Oncology Bioscience, Research & Development (R&D), AstraZeneca, Waltham, MA, United States

**Keywords:** age, immunotherapy, CT26, OX40, PD-L1, CTLA-4

## Abstract

**Introduction:**

Immuno-oncology (IO) research relies heavily on murine syngeneic tumor models. However, whilst the average age for a cancer diagnosis is 60 years or older, for practical purposes the majority of preclinical studies are conducted in young mice, despite the fact that ageing has been shown to have a significant impact on the immune response.

**Methods:**

Using aged (60-72 weeks old) mice bearing CT26 tumors, we investigated the impact of ageing on tumor growth as well as the immune composition of the tumor and peripheral lymphoid organs.

**Results:**

We found many differences in the immune cell composition of both the tumor and tumor-draining lymph node between aged and young mice, such as a reduction in the naïve T cell population and a decreased intratumoral CD8/Treg ratio in aged animals. We hypothesized that these differences may contribute to impaired anti-cancer immune responses in aged mice and therefore assessed the anti-tumor efficacy of different IO therapies in aged mice, including both co-stimulation (using an anti-OX40 antibody) and immune checkpoint blockade (using anti-PD-L1 and anti-CTLA-4 antibodies). Whilst aged mice retained the capacity to generate anti-tumor immune responses, these were significantly attenuated when compared to the responses observed in young mice.

**Discussion:**

These differences highlight the importance of age-related immunological changes in assessing and refining the translational insights gained from preclinical mouse models.

## Introduction

Over half of cancers arise in individuals over the age of 60 (1) and yet the majority of preclinical studies are undertaken in comparatively young mice, typically 6-8 weeks old. These murine models are invaluable for evaluating the fundamental biology of a particular target or for delineating the mechanism of action of candidate drugs. However, these preclinical models frequently overpredict efficacy and have so far proven to be of limited predictive value to the clinical setting (2). This may be partly due to their failure to recapitulate many of the age-induced changes that impact the immune system and therefore potentially the tumor microenvironment (TME) and the efficacy of cancer immunotherapy in patients (2).

Indeed, ageing is accompanied by a progressive deterioration of the immune system, frequently termed immunosenescence, which is the result of changes intrinsic to the hematopoietic system coupled with age-associated perturbations to peripheral tissues and secondary lymphoid organs (3, 4). A principal contributor to this process is thymic involution, which drives a decline in thymic output and a greater reliance upon cytokine and (self-) antigen-driven homeostatic proliferation to maintain the peripheral T cell pool (5). Over the lifetime of an individual, this reliance upon tonic stimulation can profoundly alter the T cell repertoire, favoring the development of memory T cell populations over the naïve T cell pool (6). The numerical decline in cells of the aged immune system is compounded by age-related inflammatory processes that occur within peripheral tissues, often collectively termed ‘inflamm-aging’ (7). These changes promote immune cell dysfunction and increased activity of suppressive immune cell subsets, such as regulatory T cells (Treg) and myeloid-derived suppressor cells (MDSCs) (8). Thus, in ageing patients these various contributors to immunosenescence and altered tissue microenvironments have a significant potential to impact anti-tumor immunity and the efficacy of cancer immunotherapy. Nevertheless, this is not modelled in preclinical studies carried out in 6-8 week old mice which, according to correlations between mouse age and human age, represent roughly the equivalent of a teenage human (9). We therefore conducted studies to evaluate the impact of age on the anti-tumor immune response and the outcome of IO therapies to assess whether recapitulating this patient characteristic in preclinical mouse models could increase their predictive value.

## Materials and methods

### Animals

Female BALB/c mice were supplied by Charles River UK at 6 to 8 weeks of age (young) or 60 to 72 weeks of age (aged). These mice were aged at Charles River UK specifically for the purpose of these studies and housing conditions were kept the same as for young mice. Mice were housed under specific pathogen-free conditions in Tecniplast Green Line Sealsafe Individually Ventilated Cages (IVC) changed weekly and holding a maximum of 6 animals with irradiated aspen chip bedding, Nestlets nesting material, a tunnel and wooden chew blocks. Mice were housed on a 12/12 light/dark cycle, at 20-24°C and 45-65% humidity with *ad libitum* UV-treated water and RM1 rodent diet (SAFE). Mice underwent a minimum of 5 days of acclimatization after arrival in the animal facility before study initiation.

### Tumor model and monitoring

The murine colon carcinoma cell line CT26 was obtained from ATCC and maintained in RPMI 1640 media supplemented with 10% FBS. It did not undergo any *in vivo* passaging and was maintained under limited passage from original stocks (typically under 5). It was reauthenticated using STR-based DNA profiling and multiplex polymerase chain reaction and tested for murine viruses and mycoplasma (IDEXX Bioresearch). For implantation, mice were shaved on the right flank and subcutaneously injected with 5 x 10^5^ CT26 cells in 100 µL PBS. Tumor volume was measured 3 times per week using electronic callipers and calculated using the formula (width^2^ × length)/2. Mice were euthanized when they reached humane welfare limits pertaining to tumor volume (average diameter of 15mm) or tumor condition (ulceration of the skin above the tumor). Tumor growth rate was calculated as previously described (10) by fitting the tumor growth curve of each animal to the exponential model log10(tumor volume) = a + b * time + error, where a and b are parameters that correspond to the log initial volume and growth rate, respectively.

### Experimental design

Mice were randomized to minimize differences in body weight and treated with either anti-mouse PD-L1 antibody mIgG1 D265A (AstraZeneca) intraperitoneally (IP) at 10 mg/kg twice weekly starting 4 days after tumor cell implantation for a total of 6 doses and/or anti-mouse CTLA-4 antibody (clone 9D9) mIgG1 (AstraZeneca) IP at 10 mg/kg twice weekly starting 7 days after tumor cell implantation for a total of 6 doses or anti-mouse OX40 antibody (clone OX86) mIgG2a (AstraZeneca) IP at 1 mg/kg 4 and 7 days after tumor cell implantation. Group sizes were determined using power analyses based on the variability of the tumor model in pilot studies.

### Flow cytometric analysis

Tumors were disaggregated using the gentleMACS™ Mouse Tumor Dissociation kit (Miltenyi Biotec). Tumor-draining lymph nodes (or matching inguinal lymph nodes in non-tumor bearing mice) were dissociated through a 70 µm nylon cell strainer. Cells were stained with a fixable viability dye (Thermo Fisher) and blocked with antibodies against murine CD16/CD32 (eBioscience) before staining with fluorescence-conjugated antibodies (**Supplementary Table S1**) in flow cytometry staining buffer with Brilliant Stain Buffer (BD Biosciences). Intracellular staining was performed using the FoxP3/Transcription Factor Staining Buffer Set (eBioscience) and cells were fixed in 3.7% formaldehyde. Counting beads (123Count eBeads; eBioscience) were added to the samples before acquisition on a BD LSRFortessa flow cytometer (BD Biosciences). Panel-specific FMO controls and single color controls were prepared for each experiment. Analysis was carried out using FlowJo (BD Biosciences). As an adjunct to conventional gating, for tumor samples the data was quality controlled using the flowAI plugin in FlowJo followed by T-distributed stochastic neighbor embedding (optSNE) dimensionality reduction and FlowSOM clustering analysis.

### Statistical methods

For time-to-endpoint analysis, groups were compared using a log-rank test in GraphPad Prism. Fold changes in tumor growth rates were compared using unpaired Mann-Whitney testing in GraphPad Prism. Cell populations and growth rates were compared using either two-way ANOVA with Sidak’s multiple comparisons testing or unpaired Mann-Whitney testing in GraphPad Prism.

## Results

### Peripheral immune changes in response to tumor growth are observed in aged mice

In this study, we sought to investigate the impact of age on the CT26 mouse syngeneic flank tumor model, which is a commonly-used preclinical model for assessment of novel anti-tumor therapies. To determine whether the characteristics of this model would be affected by age, we implanted CT26 tumor cells simultaneously in both aged (60-72 weeks of age) and young (6-8 weeks of age) female BALB/c mice. Correlations between mouse and human age suggest that this would correspond to 50-70 year-old and 15 year-old patients, respectively (9), and we confirmed that several hallmarks of ageing were observed in these mice at 60-72 weeks of age, such as thymic involution and increased adiposity (**Supplementary Figure 1**) (11, 12).

**Figure 1:**
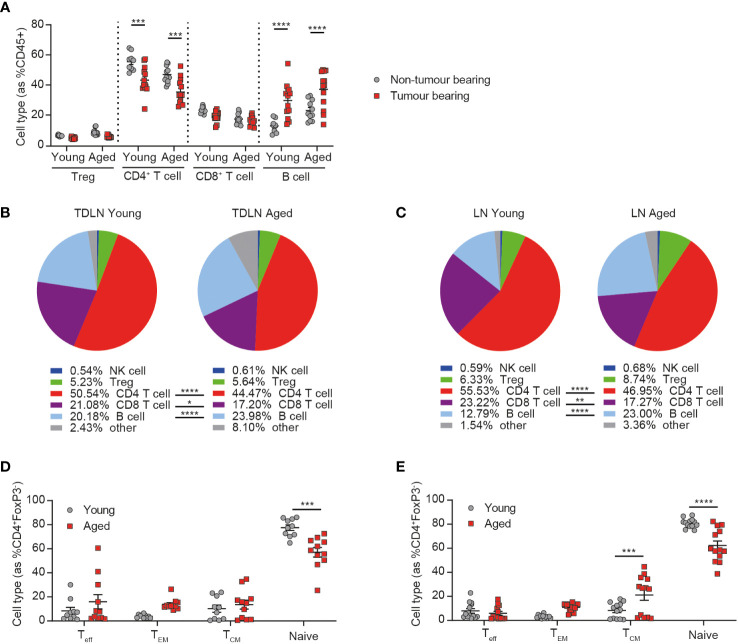
Peripheral immune changes in response to tumor growth are observed in aged mice. Inguinal lymph nodes from female BALB/c mice either at 6-8 weeks old (young) or at 60-72 weeks old (aged) were sampled in the absence or presence of CT26 tumor cells subcutaneously implanted on the flank 15-17 days prior. **(A)** Immune cell subsets within inguinal lymph nodes of tumor-bearing mice compared to non-tumor bearing mice in both young and aged mice. 5-8 mice per group. **(B)** Comparison of the immune cell subsets within inguinal tumor-draining lymph nodes (TDLN) of young (left) and aged (right) mice bearing CT26 flank tumors. 18-19 mice per group. **(C)** Comparison of the immune cell subsets within inguinal lymph nodes of young (left) and aged (right) mice in the absence of a tumor. 9-11 mice per group. **(D, E)** Frequency of CD44^-^CD62L^-^ effector (T_eff_), CD44^+^CD62L^-^ effector memory (T_EM_), CD44^+^CD62L^+^ central memory (T_CM_) and CD44^-^CD62L^+^ naïve cell subsets within the CD4^+^ T cell compartment in the inguinal lymph node **(D)** or TDLN **(E)** of both young and aged mice. The results include data from 2-3 experiments. *P < 0.05, **P < 0.01, ***P < 0.001 and ****P < 0.0001.

Given the known impact of age on the immune system, we first sought to determine whether aged animals were able to respond to the presence of a tumor by evaluating the changes triggered in the inguinal lymph node (the lymph node closest to the site of flank tumor implantation) after the addition of a tumor. We observed similar changes triggered in response to the tumor in both aged and young mice, namely a decrease in the frequency of CD4^+^ T cells and an increase in the frequency of B cells (**Figure 1A**), with the latter being a phenomenon previously described after the implantation of tumor cells (13).

Despite this similar response to the presence of a tumor, we found that the immune composition of the tumor-draining lymph node was still markedly different between young and aged mice, with a reduced frequency of both CD4^+^ and CD8^+^ T cells and an increased frequency of B cells in the aged cohort (**Figure 1B**). Similar differences were also seen in the lymph nodes of non-tumor bearing mice (**Figure 1C**) and in the spleens of aged mice, irrespective of tumor presence (**Supplementary Figures 2A, B**), suggesting that these differences are mainly driven by baseline differences in the immune status between young and aged mice, rather than a difference in the response elicited by the tumor. We also further analyzed the phenotype of CD4^+^ T cells and found that, consistent with known changes in immune homeostasis with ageing (3), the proportion of naïve T cells was significantly decreased in the inguinal lymph nodes (**Figure 1D**) and spleens (**Supplementary Figures 2C, D**) of aged mice. This decrease was also observed in tumor-draining lymph nodes, as well as increases in the central memory subset in the aged mice (**Figure 1E**). Taken together these findings suggest that the immune system of aged mice is able to respond to the presence of a tumor but that this response does not restore their immune phenotype to that seen in young mice, suggesting a potential for differential responses to immunotherapy in aged animals.

### The tumor immune infiltrate is significantly altered in aged mice

**Figure 2:**
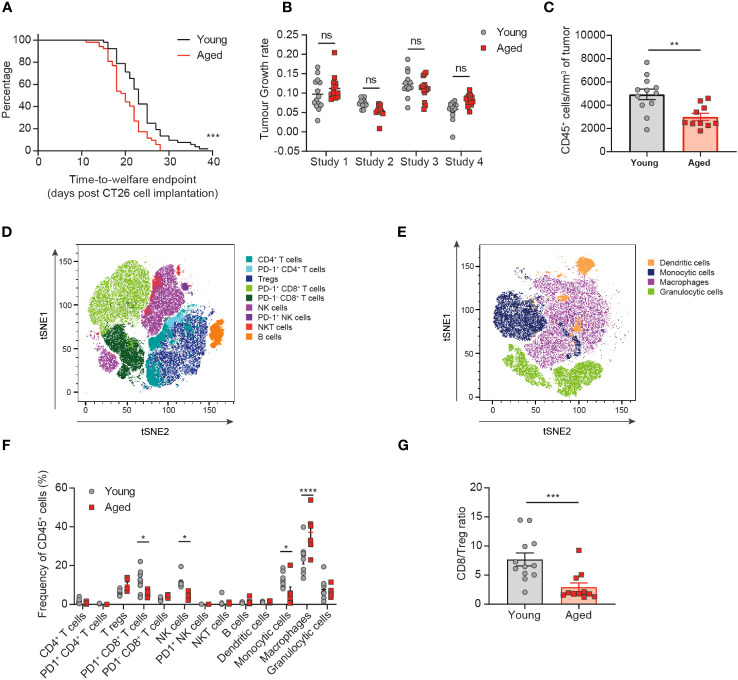
The tumor immune infiltrate is significantly altered in aged mice. CT26 tumor cells were subcutaneously implanted on the flank of female BALB/c mice either at 6-8 weeks old (young) or at 60-72 weeks old (aged). **(A)** Kaplan-Meier curves of pooled data from 4 independent experiments showing time-to-welfare endpoint of young and aged mice. 52 mice per group. **(B)** Rate of tumor growth for each animal across 4 experiments. 10-14 mice per group. **(C-G)** Flow cytometric analysis of the tumor immune infiltrate 15-17 days after tumor implantation. **(C)** Absolute cell count per mm^3^ of tumor of CD45^+^ immune cells in aged and young mice. 10-12 mice per group. **(D, E)** tSNE plot showing clusters of lymphoid **(D)** and myeloid **(E)** cell types identified by unsupervised clustering analysis. **(F)** Comparison of the proportion of immune cell clusters within the tumor of young and aged mice. 5-8 mice per group. **(G)** Comparison of the ratio of CD8^+^ T cells to Tregs in the tumor of young and aged mice. 11-12 mice per group. The results in panels **(A, B)** include data from 4 experiments, the results in panels **(C, G)** include data from 2 experiments, the results in panels **(D-F)** include data from 1 representative of 2 experiments. ns, non-significant, *P < 0.05, **P < 0.01, ***P < 0.001 and ****P < 0.0001.

Having observed a similar immune response elicited by the tumor despite baseline differences in the immune compartment of the aged mice compared to young mice, we next assessed whether age would impact the tumor immune cell infiltrate. We found that aged mice implanted with CT26 tumors reached welfare endpoints (a function of both tumor volume and tumor condition) faster than the young mice, with a median time-to-endpoint of 20 days for the aged mice versus 23 days for the young mice (**Figure 2A**, p=0.0004). However, analysis of the tumor growth rates of individual animals across 4 independent experiments revealed no consistent difference in the kinetics of tumor growth between aged and young mice (**Figure 2B**, **Supplementary Figure 3**). The CT26 tumor model is prone to develop ulceration of the skin above the tumor, which can lead to sacrifice of the animal for welfare reasons. We found that the proportion of animals being sacrificed due to skin ulceration at tumor volumes under 500mm^3^ was higher in aged mice compared to young mice (25% versus 15%, respectively; data not shown), suggesting that the difference detected in the time-to-endpoint analysis is driven by differences in tumor condition rather than tumor volume.

Having determined that CT26 tumors grew similarly in aged and young mice, we next assessed the impact of age on the immune cell composition of the tumors. Flow cytometric analysis of tumor-infiltrating immune cells revealed a significant decrease in the size of the CD45^+^ immune cell infiltrate (expressed as the absolute cell count per mm^3^ of tumor) in aged mice (**Figure 2C**, p=0.0034). Unsupervised clustering analysis then enabled the detection and quantification of several lymphoid (**Figure 2D**) and myeloid (**Figure 2E**) cell subsets within the TME (**Supplementary Figure 4**). Significant differences were observed with tumors in the aged mice containing fewer NK cells, PD1^+^ CD8^+^ T cells and monocytic cells (all p<0.05) as well as increased macrophages (p<0.0001) and a trend to increased Treg (p=0.0557) (**Figure 2F**). Altogether these changes in the tumor-infiltrating immune cell composition would suggest the potential for dampened anti-tumor immune responses in the aged mice. Indeed, we observe a reduction in key cytotoxic cell types (CD8^+^ T cells and NK cells) that have been linked to improved prognosis in several cancer types and an increase in key immunosuppressive cells types (Treg and macrophages) that have been linked overall to poorer prognosis (14). These differences in the composition of the tumor immune infiltrate also resulted in a significant decrease in the ratio of CD8^+^ T cells to Treg in the tumors of aged mice (**Figure 2G**, p=0.0007), a variable known to correlate with responsiveness to immuno-oncology therapies (15).

### The anti-tumor efficacy of anti-OX40 antibody treatment is attenuated in aged mice

**Figure 3:**
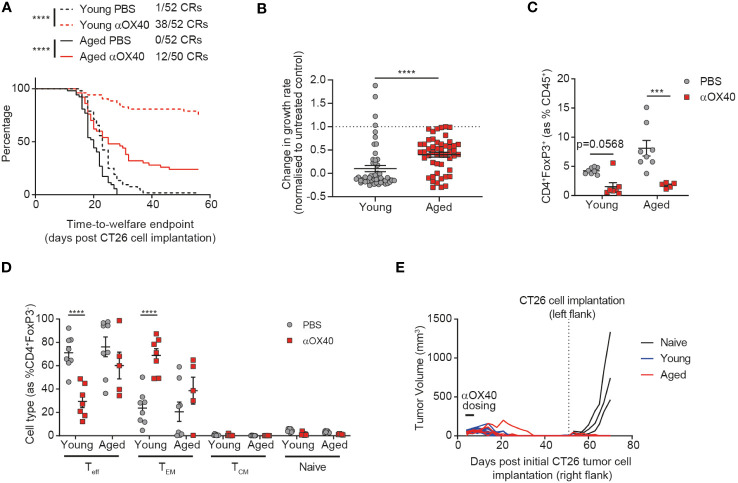
Age attenuates the anti-tumor efficacy of anti-OX40 antibody treatment. CT26 tumor cells were subcutaneously implanted on the flank of female BALB/c mice either at 6-8 weeks old (young) or at 60-72 weeks old (aged). The mice were treated with anti-OX40 antibody IP at 1 mg/kg 4 and 7 days after tumor cell implantation. **(A)** Kaplan-Meier curves showing time-to-welfare endpoint. 50-52 mice per group. **(B)** Fold change in the rate of tumor growth for each animal for both young and aged mice after anti-OX40 antibody treatment compared to untreated age-matched mice. 50-52 mice per group. **(C)** 15 days after implantation, tumors were analyzed by flow cytometry. Comparison of the proportion of Tregs in the tumor of young and aged mice after anti-OX40 antibody treatment compared to untreated mice. 5-8 mice per group. **(D)** Frequency of CD44^-^CD62L^-^ effector (T_eff_), CD44^+^CD62L^-^ effector memory (T_EM_), CD44^+^CD62L^+^ central memory (T_CM_) and CD44^-^CD62L^+^ naïve cell subsets within the CD4^+^ T cell compartment in the tumor of both young and aged mice after anti-OX40 antibody treatment compared to untreated mice. 5-8 mice per group. **(E)** 53 days after initial tumor implantation, 11 young mice and 4 aged mice that achieved complete tumor clearance after treatment with anti-OX40 antibody were re-implanted with CT26 tumor cells on the opposite flank and monitored for tumor growth. 3 control young mice (untreated and not previously tumor-bearing) were implanted as controls (naïve). The results in panels A and B include data from 4 experiments, the results in panels **(C, D)** include data from 1 representative of 2 experiments. ***P < 0.001 and ****P < 0.0001.

Having identified age-related differences in both the tumor-draining lymph node and tumor-infiltrating immune cells, we sought to investigate whether these changes would impact the response of aged mice to immunotherapy. We treated both aged and young animals with an anti-OX40 antibody and found that whilst time-to-endpoint was prolonged in both cohorts, the complete response rate was more than halved from 73% to 24% in aged mice (**Figure 3A**). This impact of age on the effect of treatment was confirmed by assessment of the tumor growth rate (**Figure 3B**, p<0.0001), with more profound anti-OX40-induced reductions in tumor growth rate observed in the young cohort, despite no age-related differences in expression of OX40 (**Supplementary Figure 5A**). Published work has previously associated the anti-tumor activity of OX40 agonism with depletion of Tregs within the TME (16) so we assessed whether the reduced efficacy of the anti-OX40 antibody in aged mice correlated with impaired Treg depletion within the tumor. We found that this was not the case and similar levels of Treg depletion were seen in the aged mice compared to the young mice (**Figure 3C**). We did not observe any impact of treatment on CD8^+^ T cell proportions (**Supplementary Figure 5B**) but we observed significant differences in the response to OX40 agonism within the CD4^+^ T cell compartment in aged mice. Whereas in young mice, treatment with the anti-OX40 antibody led to a reduction in the frequency of effector CD4^+^ T cells and an increase in effector memory CD4^+^ T cells, these changes were not seen in the aged mice after treatment (**Figure 3D**). Given the reduced induction of effector memory CD4^+^ T cells in the aged mice, we next investigated whether a memory response could also be generated after treatment. Both aged and young mice that had achieved complete tumor clearance after treatment with anti-OX40 antibody were rechallenged with CT26 tumor cells on the opposite flank 53 days later. We observed that growth of the second tumors was prevented in both aged and young mice, suggesting that memory responses had been successfully generated in the aged mice (**Figure 3E**).

### CTLA-4 blockade is less effective in aged mice

**Figure 4:**
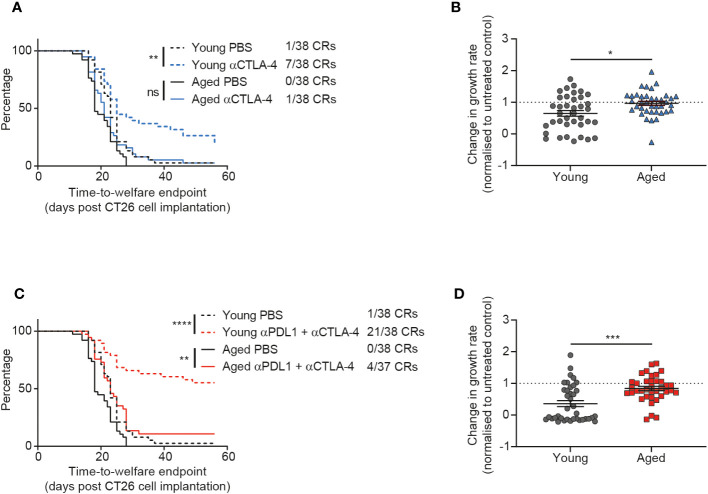
CTLA-4 blockade is less effective in aged mice. CT26 tumor cells were subcutaneously implanted on the flank of female BALB/c mice either at 6-8 weeks old (young) or at 60-72 weeks old (aged). **(A, B)** The mice were treated with anti-CTLA-4 antibody IP at 10 mg/kg twice weekly starting 7 days after tumor cell implantation for a total of 6 doses. **(A)** Kaplan-Meier curves showing time-to-welfare endpoint. 38 mice per group. **(B)** Fold change in rate of tumor growth for each animal in both young and aged mice after anti-CTLA-4 antibody treatment compared to age-matched untreated mice. 38 mice per group. **(C, D)** The mice were treated IP at 10 mg/kg twice weekly for a total of 6 doses with a combination of anti-PD-L1 antibody (starting 4 days after tumor cell implantation) and anti-CTLA-4 antibody (starting 7 days after tumor cell implantation). **(C)** Kaplan-Meier curves showing time-to-welfare endpoint. 37-38 mice per group. **(D)** Fold change in rate of tumor growth for each animal in both young and aged mice after anti-PD-L1 and anti-CTLA-4 antibodies compared to age-matched untreated mice. 37-38 mice per group. The results in panels **(A, B)** include data from 3 experiments and the results in panels **(C, D)** include data from 4 experiments. ns, non-significant, *P < 0.05, **P < 0.01, ***P < 0.001 and ****P < 0.0001.

Given the impact of age on the anti-tumor efficacy of the immune agonist OX40 treatment, we next sought to determine how age might affect the response to immune checkpoint blockade. We investigated CTLA-4 blockade and saw a significant impact of age on treatment response. Whereas young mice displayed a prolonged time to endpoint (p=0.0023) and a 18% complete response rate after treatment with anti-CTLA-4 antibody, this was not the case in the aged mice and we saw a reduced complete response rate of only 3% (**Figure 4A**). This dampening in the effect of CTLA-4 blockade in aged mice was also seen in the tumor growth rate analysis, where the fold change in growth rate compared to the untreated group was significantly less pronounced in the aged mice (**Figure 4B**, p=0.0104).

In clinical practice, it has been observed that addition of PD-1 or PD-L1 blockade to CTLA-4 inhibition can increase the response rate and durability of responses in patients (17–19). Therefore we assessed whether addition of an anti-PD-L1 antibody could overcome the reduced responsiveness of aged mice to CTLA-4 blockade. When we first assessed PD-L1 blockade monotherapy, we saw only modest anti-tumor activity and did not see a clear impact of age on response to this treatment (**Supplementary Figure 6**). However, after treatment with the combination of anti-PD-L1 and anti-CTLA-4 antibodies we saw striking age-related differences in activity of this immune checkpoint blockade regimen. Indeed, in young mice this combination led to strong anti-tumor efficacy with a 55% complete response rate (**Figure 4C**). In contrast, the complete response rate was only 11% in aged mice after treatment with the combination of anti-PD-L1 and anti-CTLA-4 antibodies (**Figure 4C**) and there was a significant decrease in the effect of the treatment on tumor growth rates (**Figure 4D** p=0.0002).

## Discussion

In this study we evaluated the impact of age, a characteristic rarely taken into account in preclinical studies, on the anti-tumor immune response in the commonly-used syngeneic CT26 flank tumor model. There is a well-recognized need to further improve preclinical mouse tumor models to better recapitulate the characteristics of cancer patient populations (2, 20, 21) and one key aspect of this population is their advanced age (1). We sought to evaluate whether accounting for this characteristic in preclinical studies would impact the outcome of the assessment of immuno-oncology treatments and potentially reduce the discrepancy between the responses achieved in preclinical studies compared to the clinical setting. To achieve this, we used mice aged between 15 and 18 months, which we found already recapitulated the thymic involution observed in older humans. Our analysis of both the tumor-infiltrating immune cells as well as secondary lymphoid organ composition in these mice also confirmed that they recapitulated the known hallmarks of immune ageing such as a reduction in the naïve T cell compartment (22).

We assessed the impact of age on the growth kinetics of the CT26 tumor model. From the literature, these age-related changes appear to be very variable, with some groups reporting faster tumor growth in aged mice (23), whilst others report slower tumor growth (24, 25). This variability may be partly due to strain and model-specific differences in the impact of ageing. In order for our experiments to be correctly powered to detect small perturbations in tumor growth kinetics that may be associated with age, we carried out a meta-analysis of 4 independent studies. From this large dataset we concluded that there was no overall impact of age on CT26 tumor growth rate, despite the variability intrinsic to individual experiments that revealed varying trends towards enhanced or decreased growth rates. Presented data from previous studies are usually the result of a limited data set where variation may contribute towards the conclusions drawn and may account for some of the discrepancies regarding the impact of age across published studies (23–27). These conclusions could also be compounded by the use of survival analyses to compare tumor growth as this time-to-endpoint readout also takes into account other welfare aspects such as tumor condition. Indeed, we found that the impact of age in these analyses may differ compared to measurement of tumor growth. This discrepancy was detected in our meta-analysis, where aged mice did come off study faster than young mice despite no difference in tumor growth rates. However we found that this could be at least in part driven by increased welfare issues in aged mice, especially higher rates and severity of skin ulceration above the tumors. Although the cause of this increase in skin ulceration has not been elucidated, we hypothesize that it could be linked to an overall decline in the wound healing response in aged mice (28), as these animals were also more prone to develop wounds due to implantation of microchips for animal identification (data not shown).

We also carried out immune profiling on the tumors of these aged mice and detected changes in both the frequency and the absolute numbers of several tumor-infiltrating immune cell subsets. We hypothesize that these could at least partially be due to baseline changes in immune composition driven by processes such as inflammaging [for example the observed increase in the proportion of macrophages, which are known to accumulate as a result of inflammaging and exert pro-tumorigenic effects (29, 30)] rather than entirely due to differences in the immune system’s recognition of the tumor. Nevertheless, these changes did not result in a significant difference in tumor growth rate in the aged mice suggesting that even if there is reduced immune control of the tumors in aged mice, this may be counterbalanced by reduced support for tumor growth, for example due to impaired angiogenesis (31). One limitation of this flow cytometric analysis of the tumor microenvironment is the lack of information about the spatial distribution of these immune cells within the tumor that can be generated with other techniques such as immunohistochemistry. This analysis would be of interest, especially as other reports have described changes in the tumor localization of T cells in the 4T1 tumor model in aged mice (32). For this study though, the high throughput and more quantitative approach of flow cytometry was preferred, enabling us to not only enumerate immune cells within the tumor, but also to more extensively phenotype them.

In this study, we found that age did alter responses to immuno-oncology therapies. Indeed, the effects of anti-OX40 antibody treatment were much reduced in aged compared to young mice. This is consistent with a previous report describing reduced efficacy of OX40 agonism in the CT26 tumor model although that study was carried out in younger (12 month old) mice and surprisingly saw less activity of OX40 agonism in 12 month old mice than we observe in 15-18 month old mice (33). We further extended this work to include an assessment of the impact of age on response to immune checkpoint blockade and here results were even more striking, with aged mice showing a greatly reduced anti-tumor effect of treatment with the combination of anti-PD-L1 and anti-CTLA-4 antibodies. Reduced efficacy of the monotherapies has been reported in the 4T1 model (32) and of the combination in the B16 model (34), although conversely others report increased efficacy of PD-1 blockade in aged mice (35). Although published reports would not suggest that this might be due to reduced expression of PD-1 or CTLA-4, as these are often observed to increase with ageing (36, 37), this was not the case in our experimental setting (**Supplementary Figure 7**) where we observed decreased levels of PD-1 and CTLA-4 on CD8^+^ T cells within the tumor. Overall, a decrease in responsiveness to immuno-oncology treatments with age is perhaps not unexpected though (38), given that ageing has been associated with immune dysregulation including both immunosenescence (39) as well as a chronic inflammatory state known as inflamm-aging (7). Specifically, ageing has been shown to lead to a decline in the naïve T cell pool (6) as well as defects in T cell priming (40). Priming is a crucial step in boosting anti-tumor immune responses with treatments such as anti-CTLA-4 antibodies (41) and is a known contributor to the increased sensitivity of the CT26 tumor model to immunotherapy, as it is a self-priming model (42). These age-related changes would be hypothesized to affect the outcomes of CTLA-4 blockade preferentially over PD-1/PD-L1 blockade based on their mechanisms of action (41) and this is reflected both in our dataset, where we did not detect an impact of age on the efficacy of PD-L1 blockade, as well as in clinical analyses of the impact of age on treatment outcomes of patients receiving PD-1/PD-L1 blocking antibodies, which do not report a decline in responsiveness to treatment associated with age (38). These types of clinical meta-analyses though mainly compare middle-aged patients against older patients (typically with a cutoff of 65 years of age), unlike our analysis where we compare very young mice (thought to represent roughly teenage patients) against aged mice (thought to represent middle aged patients). We suggest that the reduced sensitivity to immune checkpoint blockade of the CT26 model grown in aged mice, with only a subset of animals displaying responses to anti-PD-L1 and anti-CTLA-4 antibodies rather than a majority of complete responses, is a closer approximation to the response rates observed in clinical practice and could indicate improved translatability (17). This work further supports the need, already described by several other groups (2, 20, 21), to better model the effects of immuno-oncology drug candidates in the setting of the aged immune system as we find that age can significantly impact response to therapeutic treatments in one of the most responsive mouse models commonly used in immuno-oncology drug discovery (33). Future work could further build on this to assess additional characteristics of cancer patients and increase the generalizability of our findings, for example by repeating the study in male mice where sex differences in the immune system could alter the impact of ageing on anti-tumor immune responses.

In conclusion, we show that aged mice represent valuable models for the preclinical assessment of immuno-oncology therapies and that they could prove more accurate than young mice in predicting the efficacy of novel treatments due to the clear impact of age on both natural immunity to cancer and the ability to effectively respond to specific immuno-oncology treatments.

## Data availability statement

The original contributions presented in the study are included in the article/**Supplementary Material**. Further inquiries can be directed to the corresponding author.

## Ethics statement

The animal study was approved by Babraham Institute Animal Welfare and Ethical Review Body, Cambridge, UK. The study was conducted in accordance with the local legislation and institutional requirements.

## Author contributions

SS: Conceptualization, Formal Analysis, Writing – original draft. JW: Writing – original draft. LP: Formal Analysis, Writing – review & editing. MM: Writing – review & editing, Supervision. VV: Writing – review & editing, Conceptualization. MR: Writing – review & editing, Supervision. RW: Writing – review & editing. SD: Writing – review & editing, Supervision.
