# Supplementary material for: Age-induced changes in anti-tumor immunity alter the tumor immune infiltrate and impact response to immuno-oncology treatments
Source: Front Immunol. 2023 Oct 18;14:1258291. doi: 10.3389/fimmu.2023.1258291 (PMC10618668; doi:10.3389/fimmu.2023.1258291)
Supplement: Supplementary file 1 [file DataSheet_1.pdf]

Supplementary Material

Age-induced changes in anti-tumor immunity alter the tumor immune infiltrate and impact response to immuno-oncology treatments

Suzanne I. Sitnikova[1*], Jennifer A. Walker[1], Laura B. Prickett[2], Natasha A. Karp[3], Michelle Morrow[1], Viia Valge-Archer[1], Matthew J. Robinson[1], Robert W. Wilkinson[1], Simon J. Dovedi[1]

[1]Early Oncology Discovery, R&D, AstraZeneca, Cambridge, UK

[2]Early Oncology Bioscience, R&D, AstraZeneca, Waltham, MA, USA

[3]Data Science & Quantitative Biology, Discovery Sciences, R&D, AstraZeneca, Cambridge, UK

*** Correspondence:**
Suzanne Sitnikova
suzanne.sitnikova@astrazeneca.com

A

TARGET	FLUOROPHORE	CLONE	SUPPLIER
CD45	BUV395	30F11	BD
CD3	FITC	17A2	BioLegend
CD19	PE	1D3/CD19	BioLegend
CD4	BV785	RM4-5	BioLegend
CD8	BUV737	53-6.7	BD
CD44	BV510	IM7	BioLegend
CD62L	BV650	MEL-14	BD
OX40	APC	OX86	BioLegend
PD-1	BV605	29F.1A12	BioLegend
NKp46	BV711	29A1.4	BioLegend
Ki67	eF450	SolA15	eBio
FoxP3	PE-eF610	FJK-16s	eBio
CTLA-4	PE-Cy7	UC10-4B9	BioLegend

B

TARGET	FLUOROPHORE	CLONE	SUPPLIER
CD45	BUV395	30F11	BD
Ly6C	BV510	HK1.4	BioLegend
Ly6G	BV650	1A8	BioLegend
CD11b	AF488	M1/70	BioLegend
F4/80	PEDazzle594	BM8	BioLegend
MHC II	BV711	M5/114.15.2	BioLegend
CD11c	BV785	N418	BioLegend
PD-L1	PE	10F.9G2	BioLegend
OX40	APC	OX86	BioLegend
PD-1	BV605	J43	eBio
CD8	BUV737	53-6.7	BD
Ki67	eF450	SolA15	eBio

BD = BD Biosciences
eBio = eBioscience/ThermoFisher Scientific

Supplementary table S1: Flow cytometry antibodies

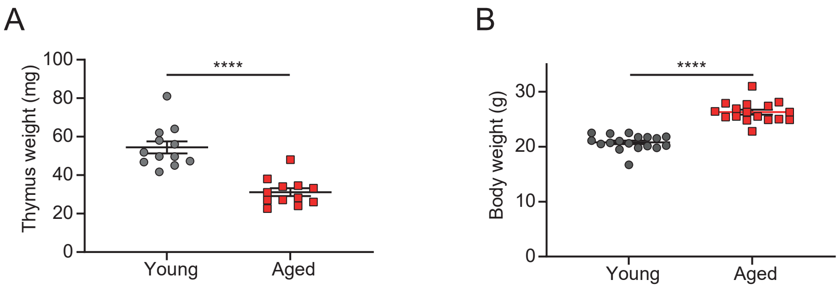

Supplementary Figure 1: Thymic involution and increased adiposity in aged mice

(A) Comparison of the thymic weight of young and aged mice. 12 mice per group. (B) Comparison of the body weight of young and aged mice. 17-18 mice per group. **** P < 0.0001.

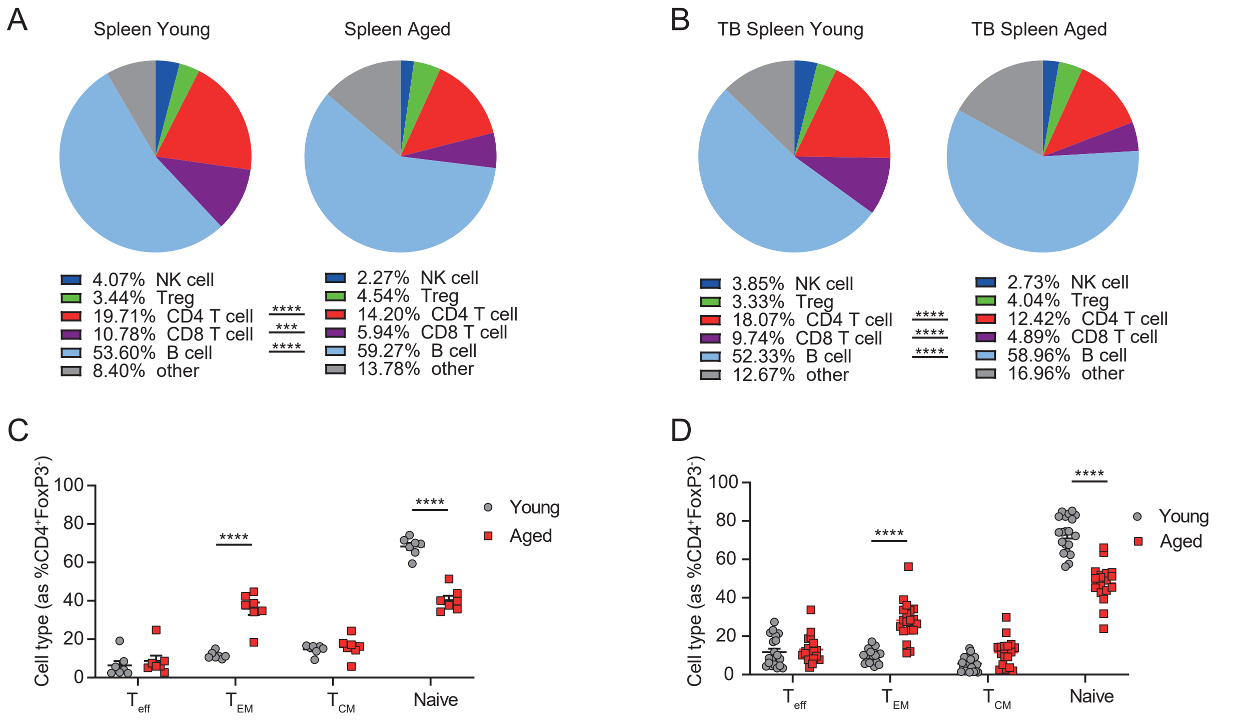

Supplementary Figure 2: Changes in splenic immune cell composition in response to tumor presence and age

Spleens from female BALB/c mice either at 6-8 weeks old (young) or at 60-72 weeks old (aged) were sampled in the absence or presence of CT26 tumor cells subcutaneously implanted on the flank 15-17 days prior. (A) Comparison of the immune cell subsets within the spleen of young (left) and aged (right) mice in the absence of a tumor. 5-8 mice per group. (B) Comparison of the immune cell subsets within the spleens of young (left) and aged (right) mice bearing CT26 flank tumors 15-17 days after implantation. 18-20 mice per group. (C and D) Frequency of $CD44^-CD62L^-$ effector (Teff), $CD44^+CD62L^-$ effector memory (TEM), $CD44^+CD62L^+$ central memory (TCM) and $CD44^-CD62L^+$ naïve cell subsets within the $CD4^+$ T cell compartment in the spleen of non-tumor bearing (C) or tumor-bearing (D) young and aged mice. 7-20 mice per group. The results include data from 1-3 experiments. ** P < 0.01, *** P < 0.001 and **** P < 0.0001.

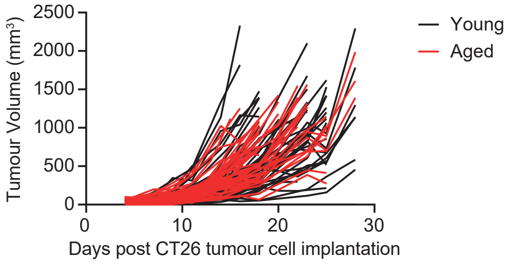

Supplementary Figure 3: Impact of age on CT26 tumor growth

CT26 tumor cells were subcutaneously implanted on the flank of female BALB/c mice either at 6-8 weeks old (young) or at 60-72 weeks old (aged). Tumor growth curves of pooled data from 4 independent experiments. 52 mice per group.

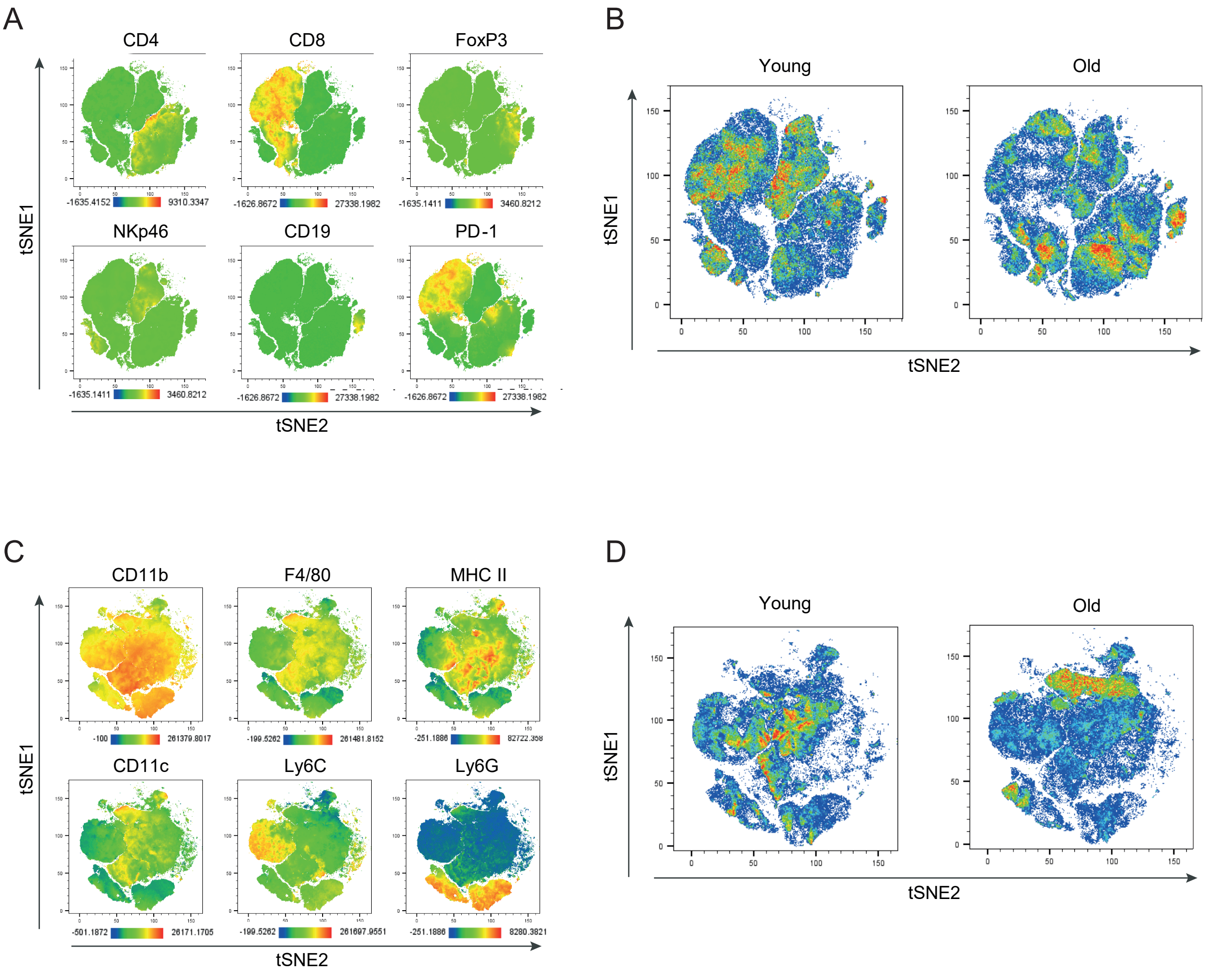

Supplementary Figure 4: Description of the tumor-infiltrating immune cell clusters

CT26 tumor cells were subcutaneously implanted on the flank of female BALB/c mice either at 6-8 weeks old (young) or at 60-72 weeks old (aged). Flow cytometric analysis of the tumor immune infiltrate was carried out 15-17 days after tumor implantation. Dimensionality reduction and unsupervised clustering analysis were applied to both a lymphoid (A and B) and a myeloid (C and D) cell-focussed analysis panel. (A) tSNE plots of each marker used to define the lymphoid cell clusters. (B) Prevalence of the various lymphoid immune cell clusters among untreated young (left) and aged (right) mice. (C) tSNE plots of each marker used to define the myeloid cell clusters. (D) Prevalence of the various myeloid immune cell clusters among untreated young (left) and aged (right) mice.

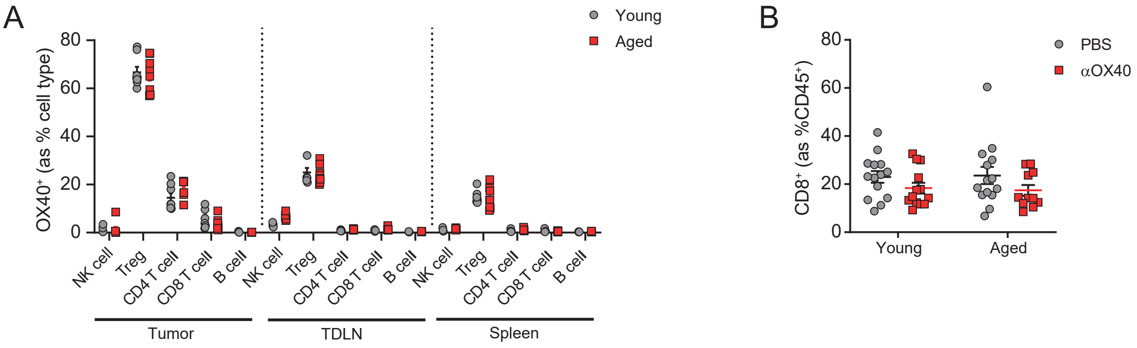

Supplementary Figure 5: Effect of anti-OX40 agonism on intratumoral CD8+ T cells

CT26 tumor cells were subcutaneously implanted on the flank of female BALB/c mice either at 6-8 weeks old (young) or at 60-72 weeks old (aged). Flow cytometric analysis of the tumor immune infiltrate, tumor-draining lymph node (TDLN) and spleen was carried out 15-17 days after tumor implantation. (A) OX40 expression on 5 immune cell types within the tumor, TDLN and spleen. 8 mice per group. (B) The mice were treated with anti-OX40 antibody IP at 1 mg/kg 4 and 7 days after tumor cell implantation. 15 days after implantation, tumors were analysed by flow cytometry. Comparison of the proportion of CD8+ T cells in the tumor of young and aged mice after anti-OX40 antibody treatment compared to untreated mice. 11-14 mice per group.

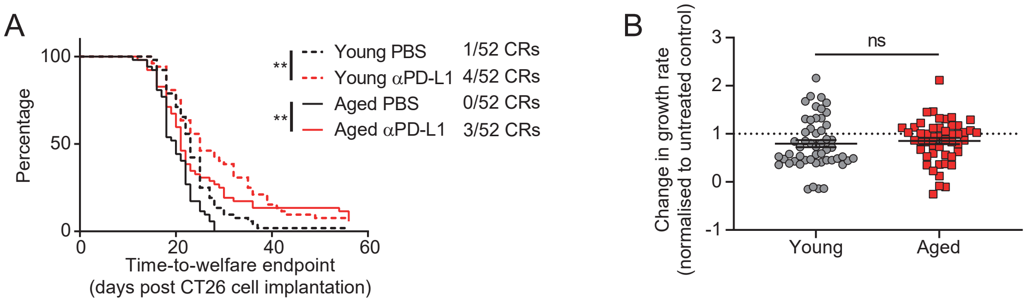

Supplementary Figure 6: Effect of PD-L1 blockade in aged mice

CT26 tumor cells were subcutaneously implanted on the flank of female BALB/c mice either at 6-8 weeks old (young) or at 60-72 weeks old (aged). The mice were treated with anti-PD-L1 antibody IP at 10 mg/kg twice weekly starting 4 days after tumor cell implantation for a total of 6 doses. (A) Kaplan-Meier curves showing time-to-welfare endpoint. 52 mice per group. (B) Fold change in rate of tumor growth for each animal in both young and aged mice after anti-PD-L1 antibody treatment compared to age-matched untreated mice. 52 mice per group. The results include data from 4 experiments. ns: non-significant, ** $P < 0.01$.

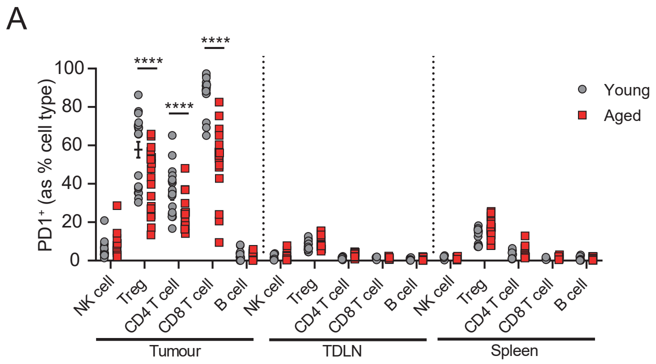

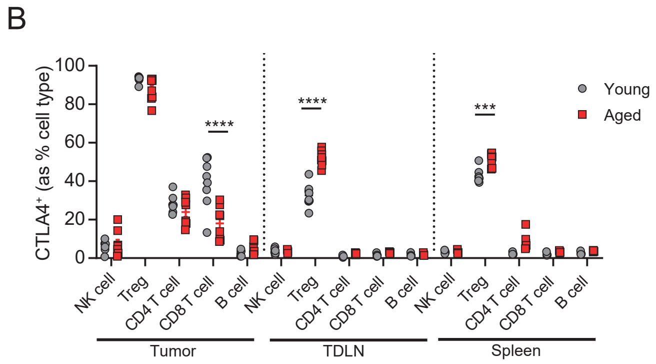

Supplementary Figure 7: Effect of age on immune cell expression of PD-1 and CTLA-4

CT26 tumor cells were subcutaneously implanted on the flank of female BALB/c mice either at 6-8 weeks old (young) or at 60-72 weeks old (aged). Flow cytometric analysis of the tumor immune infiltrate, tumor-draining lymph node (TDLN) and spleen was carried out 15-17 days after tumor implantation to quantify PD-1 (A) or CTLA-4 (B) expression on 5 immune cell types. 8-20 mice per group.
